# Une cellulite cervicale révélant un carcinome extensif de la thyroïde

**DOI:** 10.11604/pamj.2019.33.77.11564

**Published:** 2019-05-30

**Authors:** Souha Kallel, Malek Mnejja

**Affiliations:** 1 Service ORL et Chirurgie Cervico-Faciale, CHU Habib Bourguiba, 3029 Sfax, Tunisie

**Keywords:** Paupières, hidrocystome, eccrine, appocrine, Cellulitis, thyroid, papillary cystic carcinoma

## Abstract

We report the case of a 45-year old woman with a 15-day history of anterior cervical swelling increasing very quickly in size associated with a 7-day history of local inflammation. Clinical examination showed voluminous tender and fixed swelling occupying all the anterior cervical region. The skin's surface was inflammed with permeation nodule (Figure 1, A). The patient was febrile at 38° with good overall physical condition Cervical ultrasound showed voluminous right laterocervical mass that, given the size, made surgical exploration difficult. CT scan showed voluminous thyroid mass, multi-cystic in the superficial tissues, compressing the trachea and the esophagus and pressing the vascular axis of the neck (Figure 1, B and C). Laboratory tests showed leukocytosis 13500 cells/ml, anemia to 8.3 g/dl, CRP 42mg/L and normal thyroid test. Fine needle aspiration biopsy showed purulent and very inflammed material without cytologic signs of malignancy. The patient underwent surgery. Surgical exploration showed several superficial cystic and haemorrhagic cubicles with, in depth, tumor tissue infiltrating the trachea. The tumor laterally invaded the right vascular axis and spread to the suprahyoid muscles, the right submandibular region and the twelfth cranial nerve. Total extended thyroidectomy was performed. Histological examination showed papillary thyroid carcinoma measuring 11cm along its longer axis. Based on our literature review, this is the first case of locally invasive papillary cystic carcinoma of the thyroid revealed by subacute infection.

## Image en médecine

C'est une femme âgée de 45 ans qui présente depuis 15 jours une tuméfaction cervicale antérieure qui a augmenté très rapidement de taille avec apparition de signes inflammatoires locaux depuis 7 jours. L'examen a trouvé une énorme tuméfaction occupant toute la région cervicale antérieure, tendue et fixe. La peau en regard était inflammatoire avec présence d'un nodule de perméation (A). La patiente était fébrile à 38° avec un état général conservé. Une échographie cervicale a montré une volumineuse masse latéro-cervicale droite d'exploration difficile vu la taille. La TDM a montré une énorme masse thyroïdienne multi-kystique en superficie, comprimant la trachée et l'œsophage et refoulant l'axe vasculaire du cou (B, C). Le bilan biologique a noté une hyperleucocytose à 13.500 éléments/ml, une anémie à 8.3 g/dl, une CRP à 42mg/l et un bilan thyroïdien normal. La cytoponction a montré un matériel purulent et très inflammatoire sans signes cytologiques de malignité. D'où la décision était d'opérer la patiente. L'exploration opératoire a trouvé plusieurs logettes superficielles de contenu kystique et hémorragique avec en profondeur du tissu tumoral infiltrant la trachée. La tumeur arrivait latéralement à l'axe vasculaire droit et en haut jusqu'au niveau des muscles supra-hyoïdiens, la région submandibulaire droite et le nerf XII. Nous avons réalisé une thyroïdectomie totale élargie. L'examen histologique a conclu à un carcinome papillaire de la thyroïde de 11 cm de grand axe. Notre revue de la littérature n'a pas trouvé de carcinome papillaire kystique localement invasif de la thyroïde et se révélant par un tableau infectieux subaigu.

**Figure 1:**
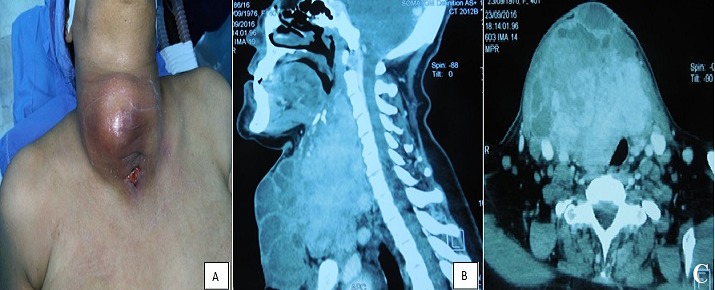
Présentation clinique: A) et scannographique; (B,C) d’un carcinome papillaire kystique extensif de la thyroïde

